# Isolated Sixth Cranial Nerve Palsy as the First Presenting Sign of MOG Antibody-Associated Disease in a Three-Year-Old Child

**DOI:** 10.22599/bioj.527

**Published:** 2026-03-02

**Authors:** Courtney Healey, Lloyd Bender, Anna Zatorska, Lucas (Tadeusz) Ginter

**Affiliations:** 1 Advanced Orthoptist, Moorfields Eye Hospital, UK; 2 Consultant Ophthalmologist, Moorfields Eye Hospital, UK; 3 Foundation Year 1 Doctor, Moorfields Eye Hospital, UK; 4 Speciality Doctor in Paediatric Neurology, St. George’s Hospital, London, UK

**Keywords:** Myelin oligodendrocyte glycoprotein antibody-associated disease (MOGAD), Multiple Sclerosis (MS), Acquired demyelinating syndromes (ADS), Sixth cranial nerve palsy, Esotropia, Paediatric Neuro-Ophthalmology, Myelin oligodendrocyte glycoprotein (MOG), Neuromyelitis Optica Spectrum Disorder (NMOSD)

## Abstract

**Background:**

Myelin oligodendrocyte glycoprotein antibody-associated disease (MOGAD) is a neurological, autoimmune, demyelinating disorder. Clinical phenotypes differ between adults and children, and MOGAD is more commonly observed in the paediatric population compared to the adult population. Cases of isolated cranial nerve palsies as initial clinical phenotypes of MOGAD are rarely reported in the literature. Here, we report a child who presented with an isolated left sixth cranial nerve palsy as the initial sign of anti-myelin oligodendrocyte glycoprotein antibody associated disease (MOGAD).

**Method:**

In this case study, we report on a three-year-old female known to the orthoptic and paediatric ophthalmology services due to an intermittent exotropia. The same child later develops an acute convergent deviation of the left eye preceded by a viral illness with no other systemic symptoms. This case reports on the clinical findings and treatment over a five-year period.

**Results:**

Immunological investigations revealed positive oligoclonal bands in CSF but negative in serum. Serology was positive for MOG antibodies. Magnetic Resonance Imaging (MRI) showed acute demyelination in the pons and cerebellum with accompanying lesions in cerebral white matter bilaterally and incidental band heterotropia. The symptomatic lesion was found to be a small plaque in the left lower pons in the intrinsic course of the left sixth cranial nerve, suggestive of acute demyelination. The patient completed a five-day course of intravenous methylprednisolone 30 mg/kg daily, followed by a reducing course of oral prednisolone.

**Follow up discussion:**

Although brain stem involvement in MOGAD is common, isolated cranial palsies are rare and are not commonly reported as presenting symptoms in children (Barr *et al*., 2000). Patients with MOG positive antibodies have relatively favourable outcomes. Here, we highlight the importance of considering MOGAD in the differential diagnosis of isolated cranial nerve palsies, particularly in the paediatric population.

## Introduction

Myelin oligodendrocyte glycoprotein antibody-associated disease (MOGAD) is an autoimmune, demyelinating disorder (Wynford-Thomas, Jacob and Tomassini, 2019). MOG is a protein expressed on the outer surface of oligodendrocytes and myelin sheath in the CNS. However, its biological role is not clear. Research has shown that MOG may act as a cellular receptor, adhesion molecule or a regulator of microtubule stability (Johns and Bernard, 1999; Marta *et al*., 2005). In MOGAD, the immune system attacks the MOG specific protein found on the myelin sheath of the central nervous system. Clinical phenotypes include optic neuritis (ON), acute disseminated encephalomyelitis (ADEM), encephalitis, and transverse myelitis (TM) (Hua *et al*., 2023; Parrotta and Kister, 2020).

The disease clinically differs from multiple sclerosis (MS) and aquaporin-4 (AQP4)-IgG-positive NMO, and current literature informs us that the disease presents with differences between the adult and paediatric population. ADEM is the most common presenting clinical phenotype in children whereas optic neuritis is the most common clinical phenotype in the adult population (Al-Ani, Chen and Costello, 2023). Santoro *et al*. (2023) also informs us that ADEM is the most common presenting phenotype in MOGAD amongst children less than five years of age, followed by ON and/or TM. In addition to this, roughly one quarter of children diagnosed with MOGAD have at least one relapse that occurs within three years of onset of disease.

MOGAD is more commonly observed in the paediatric population compared to that identified in the adult population (Rinaldi *et al*., 2021; Yılmaz, 2024) and the disease course has shown to be either monophasic or relapsing (Jarius *et al*., 2018). Cranial nerve palsies are rarely reported in the literature as a clinical phenotype of MOGAD within the paediatric population, however there are some reports in adulthood. Ng *et al*. (2024) describes a case of a thirty-eight-year-old female presenting with a right sixth cranial nerve palsy and nystagmus, who is confirmed to have MOGAD. Despite treatment of intravenous methylprednisolone, intravenous immunoglobulin and plasma exchange, the study reports only partial neurological improvement.

In the paediatric cohort of patients, sixth cranial nerve palsies are more likely due to neoplasms, trauma and intracranial hypertension as a false localising sign (Harley, 1980; Merino *et al*., 2010; Park *et al*., 2019). Gaccon (2006) describes a case of a two-year-old child, presenting with a sudden onset esotropia and papilloedema who, at the time, was diagnosed with childhood of multiple sclerosis. The orthoptic assessment reports full eye movements and no evidence of a sixth cranial nerve palsy.

There have been few reports of a cranial nerve palsy as the first presenting sign of MOGAD, in the paediatric population (Du *et al*., 2022). Cobo-Calvo *et al*. (2019) describes a case of a 16-year-old who presented with retro-orbital pain and a bilateral third cranial nerve palsy, the patient was subsequently found to be MOG-IgG positive. Du *et al*. (2022) present a review of various cranial nerve palsies as the first presenting sign of MOGAD, however, to our knowledge, this is the first report of an isolated sixth cranial nerve palsy as the first presenting sign of MOGAD. In this case study, we report the details of a three-year-old child who presented with an isolated left sixth cranial nerve palsy as the initial clinical phenotype of MOGAD.

### Case presentation

Our patient was a three-year-old multiracial female, born at 37 weeks, whose birth history was complicated by maternal gestational diabetes mellitus and maternal infection. The patient underwent intravenous antibiotics and was admitted to the neonatal unit but did not require intubation. At presentation, she had reached all her developmental milestones and was up to date with all vaccinations.

The patient had been under the orthoptic and paediatric ophthalmology service since 2019, when the patient was 2 years old (or 27 months of age). In 2019, following a fall at a playground, the child’s mother noticed a drift of the left eye outwards (exotropia). Examination showed an intermittent left exotropia, with full range of eye movements and some binocular function including proven motor fusion. Stereopsis was not possible to prove in 2019 due to age and co-operation. Vision was assessed using Cardiff Acuity Cards at 1 metre and was found to be 0.2 LogMAR (6/9.5 Snellen) in each eye. Pupils were equal and reactive to light and there was no relative afferent pupillary defect. Dilated fundoscopy was normal with no evidence of optic nerve head swelling. The Paediatric Ophthalmology team had planned to review the patient in early 2020, however due to the Coronavirus disease (COVID-19) pandemic the patient’s appointment was postponed. Two telephone consultations were scheduled during the pandemic and the patient’s mother reported no concerns and stated that she rarely noticed the exotropia.

In April 2021 the patient’s mother contacted the department urgently following concerns that the exotropia had recurred (seventeen months since the patient was last seen face to face by the Ophthalmology team). This was preceded by a viral illness with no other systemic symptoms. According to her mother, the patient did not have any headaches, nausea, vomiting, fever, weight loss, night sweats, and was otherwise fit and well. The patient was now 3 years old (46 months of age).

Orthoptic findings showed visual acuity of 0.100 LogMAR in the right eye and 0.260 LogMAR in the left eye unaided (Crowded Kays Pictures at 3 m). There was no relative afferent pupillary defect. Cover test revealed a moderate left esotropia with diplopia for near fixation and a marked left esotropia with diplopia for distance fixation. Measurements on alternate prism cover test were 30 dioptres base out for near fixation and 50 dioptres base out for distance fixation. Ocular movements showed a –4 limitation of the left eye in abduction. Prism cover test on lateral gaze was not possible. Fundoscopy findings were unremarkable. No papilloedema was noted. There was no palpebral fissure changes observed. There was no nystagmus noted. An assessment of visual fields and colour vision was not possible. There were no other evident neurological abnormalities and no features of encephalopathy. We subsequently referred her to the paediatric team for further investigation.

## Investigations

### Pertinent Laboratory Investigations

▪ Cerebrospinal fluid (CSF): opening pressure elevated at 34 cm H2O. Oligoclonal bands were positive in CSF but negative in serum. Cytology and chemistry were normal. Viral PCR negative
▪ Autoimmune serology: positive MOG serum antibodies, AQP4 antibody negative.
▪ Serology and serum PCR was negative for common likely pathogens.

### Magnetic Resonance Imaging (MRI) of the brain with contrast showed two unrelated pathologies

**Figure 1:**
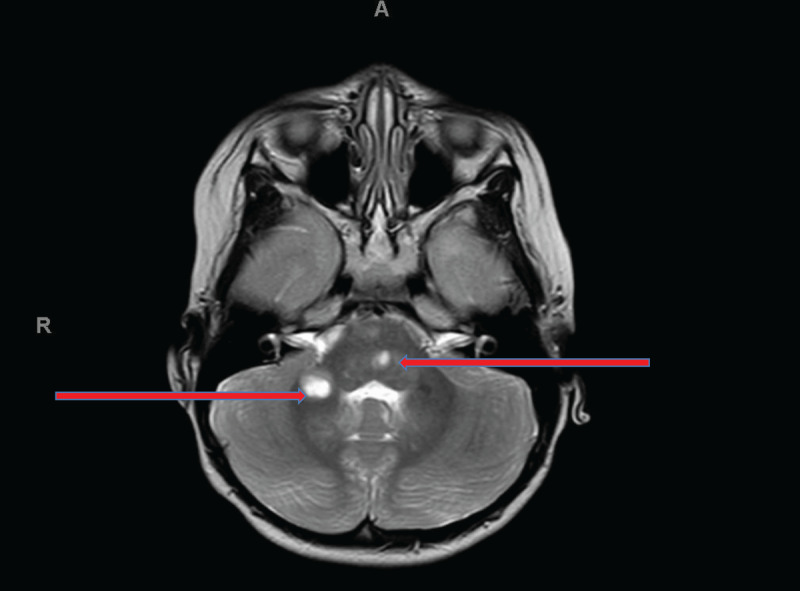
Small plaque in the left lower pons in the intrinsic course of the left sixth nerve (higher red arrow). There is an adjacent slightly larger lesion in right cerebellar white matter (lower red arrow). Both have a ‘cyst like’ appearance.

**Figure 2:**
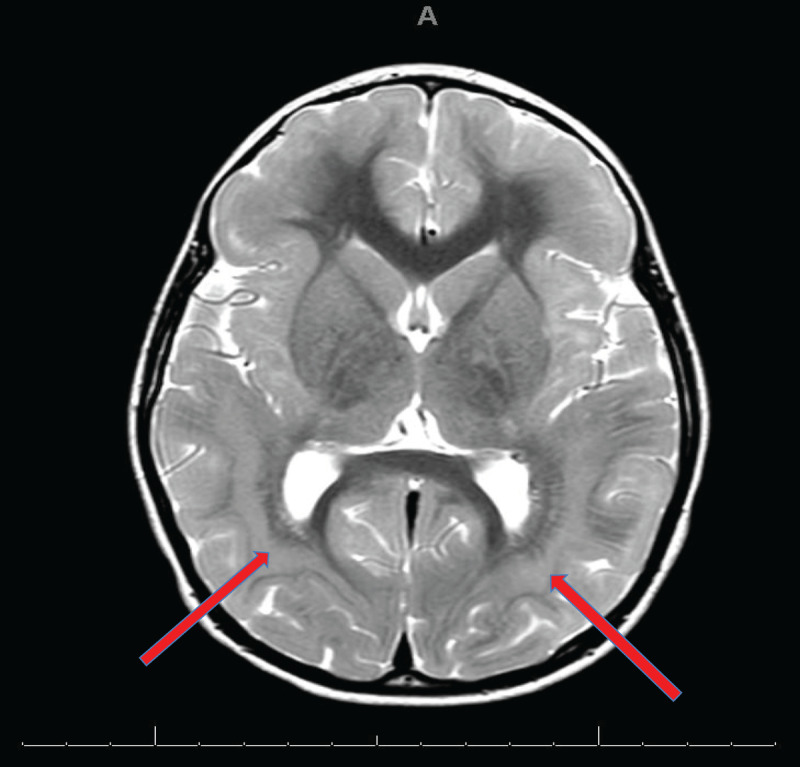
Band heterotropia as indicated by arrows.

▪ an acute demyelination in the pons and cerebellum with accompanying lesions in cerebral white matter bilaterally (Figure 1)
▪ incidental band heterotropia (Figure 2). This is a radiological feature due to arrested neuronal migration resulting in subcortical bands. The effect of this arrested neuronal migration is variable and can range from normal intelligence with seizures later in life to severe intellectual disability and early onset seizures.

### Working diagnosis and treatment

The patient was diagnosed with anti MOG antibody associated disease. She completed a five-day course of intravenous Methylprednisolone (30 mg/kg), followed by a reducing course of prednisolone starting at 2 mg/kg/day. The cerebrospinal fluid (CSF) pressure was reduced to 21 cm H20 during the lumbar puncture procedure. Of note, our patient had an incidental finding of band heterotopia (rare cortical malformation) which may manifest in cognitive impairment and seizures. The patient did not have features of cognitive impairment or a history or diagnosis of seizures. However, she was referred to paediatric neurology and the genetics team for follow up.

Five days post intravenous Methylprednisolone (30 mg/kg) treatment, the patient was assessed by the Orthoptist on the ward. Visual acuity was unaided: 0.100 LogMAR in the right eye and eye 0.340 LogMAR in the left eye (Crowded Kays Pictures at 3 m), unable to use a pinhole. There was no relative afferent pupillary defect. Cover test revealed a marked left esotropia with diplopia for near fixation and a marked left esotropia with diplopia for distance fixation. Measurements on alternate prism cover test were 40 dioptres base out for near fixation and 55 dioptres base out for distance fixation. Ocular movements showed a –4 limitation of the left eye in abduction.

Consultant assessment reported that fundoscopy findings were unremarkable. No papilloedema was noted.

### Outcome and follow-up

The patient was reviewed by the Orthoptist and Ophthalmologist four weeks later. Visual acuity was equal in both eyes (0.080 LogMAR Crowded Kays Pictures at 3 m). Cover test revealed a slight left esotropia for near and a small left esotropia for distance, with diplopia. Measurements on alternate prism cover test were 12 dioptres base out for near fixation and 16 dioptres base out for distance fixation. Ocular movement testing showed a –1.5 limitation of the left eye in abduction, indicating improvement of the left lateral rectus function. There was no disc swelling. The plan was for the patient to be followed up by a paediatric neurologist with input from the genetics team.

A subsequent cortical malformation gene panel identified an error in the gene DCX. A genetic diagnosis of X-linked subcortical laminal heterotopia due to fault in DCX gene (de novo) was made. This was thought not to be related to the presentation.

### Long term follow-up

After the initial diagnosis of MOGAD in April 2021, the patient had two episodes of relapse. The first relapse of the disease in December 2021 presented with optic neuritis. At this time MRI showed additional, new non-enhancing lesions in the brain and previously detected lesions from 2021 were described as less hyperintense. The right optic nerve was mildly swollen. The second relapse, in October 2024, was identified following dizziness, ataxic gait and limb weakness, MRI revealed two new plaques in the brain. Following each relapse, the patient was treated under the care of the paediatric neurology team with intravenous Methylprednisolone, followed by a reducing course of oral prednisolone. In November 2024, the patient was commenced on immunosuppressant mycophenolate mofetil (MMF), which has been shown to prevent relapse in patients with MOGAD (Li *et al*., 2020). Despite the relapse in disease over the years, the most recent orthoptic report in March 2025 showed a normal level visual acuity in each eye (unaided 0.00 LogMAR Letters at 3 m), full colour vision, full eye movements and normal binocular responses including stereopsis.

## Discussion

Our patient presented with an acute onset isolated left sixth cranial nerve palsy to the orthoptics service. Aetiologies of underlying sixth cranial nerve palsy in children include neoplasms, trauma, infectious disorders, increased intracranial pressure, and congenital causes (Osigian, Chang and Cavuto, 2017).

We cannot disregard that this patient had raised intracranial pressure at presentation (34 cm H2O). Thus, a potential causal or contributing factor to the patients sixth cranial nerve palsy. However, it seems more likely that the lesion location identified on the MRI scan caused the cranial nerve palsy identified in this case. The patient’s symptomatic lesion was found to be an enhancing small plaque in the left lower pons in the intrinsic course of the left sixth cranial nerve, suggestive of acute demyelination. Although brain stem involvement in MOGAD is common, isolated cranial nerve palsies are rare and are not commonly reported in children (Barr *et al*., 2000). Interestingly Teksam *et al*. (2016) in a study of 14 paediatric patients, describes a case of a 16-year-old male who presented with paraesthesia in the left arm and leg with accompanying diplopia. On examination, he had a right sixth cranial nerve palsy as well as reported non-specific limitations of elevation and depression in the right eye. His MRI showed contrast enhancing lesions in the right pontomedullary junction and subsequently he was diagnosed with multiple sclerosis. Similarly, Park *et al*. (2019) reported that out of 35 patients with sixth cranial nerve palsy, only one was later diagnosed with multiple sclerosis, further highlighting the rare incidence of acute demyelinating syndromes as a causative aetiology of these presentations. Neuroimaging such as MRI together with laboratory investigations play an important role in correlating clinical presentation of acute demyelinating syndromes with neuropathology. Thus, adequate and prompt referrals are key in establishing the underlying cause.

In our case study, further investigations identified the presence of serum MOG antibodies, confirming an anti-MOG antibody associated disorder and not MS. MS is not antibody driven in the same way as MOGAD (Sechi *et al*., 2022). Children with MOG positive associated disease have relatively favourable outcomes including lower disability burden than children with aquaporin-4 (AQP4) antibody associated disease or MS (Hacohen *et al*., 2017). Further, approximately 7% to 50% of patients with MOG antibodies have a monophasic course of the disease and may not suffer further relapses (Cobo-Calvo *et al*., 2021; Waters *et al*., 2020). Starting treatment with intravenous glucocorticoids urgently is recommended as this may prevent residual complications. In our case, the patient demonstrated two relapses since initial diagnosis and continues to be followed up closely by the ophthalmology, paediatric, neurology, and genetics teams.

Initial presentation of the disease showed elevated intracranial pressure of 34 cm H20. Nguyen *et al*. (2023), tells us, that raised intracranial pressure can be observed in the paediatric cohort of patients presenting with MOGAD. In this retrospective study, 20.9% were identified as having raised intracranial pressure as well as acute disseminated encephalomyelitis (ADEM). Having increased intracranial pressure and ADEM was associated with longer hospital admission and long-term neurological disability. Chaudhuri *et al*. (2022), also describes a case of an older patient aged 19 who presents with increased intracranial pressure and bilateral papilloedema. The patient is investigated and diagnosed with MOGAD. The paper discusses the suspicion of MOGAD, which clinicians should consider, as a cause of isolated increased intracranial pressure.

The case we present revealed elevated intracranial pressure in the absence of papilloedema or any other reported optic nerve anomalies. Shaw *et al*. (2023) and Lee *et al*. (2017) explain that the absence of papilloedema can be a false negative sign for raised intracranial pressure. Lee *et al*. (2017) goes on to explain that papilloedema is more common in older children than in younger children in the presence of raised intracranial pressure. Explanations include open fontanelles in younger cohort of the paediatric group and time lag for papilloedema to develop. Due to this, as clinicians, we should not rule out raised intracranial pressure in the absence of papilloedema.

To date, there have been no randomised controlled trials comparing preventative treatment for anti-MOG antibody associated disease and current practice varies based on clinical opinion and retrospective data. Regular follow up is required to monitor for further episodes of disease relapse, as patients may require long term treatment with immunosuppressive agents.

### Learning points/take home messages

▪ A sixth cranial nerve palsy may be the only presenting sign in children with MOGAD. Urgent paediatric neurology referral is warranted.
▪ MOGAD should be considered as a differential diagnosis in a case of unilateral cranial nerve palsy.
▪ Careful attention should be made to an MRI requested in a child with an isolated sixth cranial nerve palsy. If suspect patterns of demyelination are present, clinicians should include MOG antibody testing within the blood test workup.
▪ Children with anti-MOG antibody associated disease have better long-term outcomes than patients with aquaporin-4 (AQP4)-IgG-positive NMO or MS.

## Supplementary Files

All supplementary files (photographs) have been included in the main body of text.
